# Evaluation of parental knowledge, attitudes and practices regarding antibiotic use in acute upper respiratory tract infections in children under 18 years of age: a cross-sectional study in Turkey

**DOI:** 10.1186/s12887-021-03020-4

**Published:** 2021-12-06

**Authors:** Aslınur Albayrak, Nazmi Mutlu Karakaş, Bensu Karahalil

**Affiliations:** 1 Faculty of Pharmacy, Department of Clinical Pharmacy, Gazi University, Ankara, Turkey; 2 Faculty of Medicine Department of Pediatrics, Gazi University, Ankara, Turkey; 3 Faculty of Pharmacy, Department of Pharmaceutical Toxicology, Gazi University, Ankara, Turkey

**Keywords:** Upper respiratory tract infections, Parents knowledge, Attitude, Practices, Turkey

## Abstract

**Background:**

Upper respiratory tract infections (URTIs) are common in children. Antibiotics still continue to be prescribed although most URTIs are of viral origin. This is inappropriate use and this unnecessary administration contributes or may cause antibiotic resistance. The problem of unnecessary antibiotic use among children is a concern for antibiotic resistance in low- and middle-income developing countries. This study aims to evaluate the knowledge and attitudes of parents of children with upper respiratory tract infections regarding antibiotic use and their antibiotic administration practices in a tertiary care hospital in Turkey.

**Methods:**

Our study is a cross-sectional survey study. It was carried out between 14 December 2020 and 1 April 2021 for parents over 18 years of age with a child under 18 years’ old who applied to the general pediatrics outpatient clinics of Gazi University Faculty of Medicine Hospital Department of Pediatrics.

**Results:**

Five hundred fifty-four parents responded to the questionnaire (93.2% rate of response). A total of 15.7% of parents stated to use antibiotics in any child with fever. 37% of parents believed that antibiotics could cure infections caused by viruses. 6.3% of parents declared that they put pressure on pediatricians to prescribe antibiotics. While 28% of the parents who thought that the use of inappropriate antibiotics would not change the effect and resistance of the treatment, 41% thought that new antibiotics could be developed continuously. 85.6% of the parents stated that they never gave their children non-prescription antibiotics when they had a high fever. 80.9% of them declared that they never used past antibiotics in the presence of a new infection.

**Conclusion:**

According to the results of our study of parents’ lack of knowledge about antibiotics in Turkey, though generally it shows proper attitude and practices. It shows that some of the restrictions imposed by the National Action Plan are partially working. However, it is still necessary to continue to inform parents, pediatricians and pharmacists about the use of antibiotics, and to be more sensitive about the prescribing of antibiotics, and if necessary, sanctions should be imposed by the state in order to prevent unnecessary antibiotic prescriptions.

## Background

Upper respiratory tract infections (URTIs) are common in children [1]. This is probably due to the vulnerability of children to URTIs [1, 2]. However, most URTIs have been shown to be of viral origin. In this case, the use of antibiotics is unnecessary and not suitable [3]. This unnecessary use of antibiotics is known to lead to antibiotic resistance [4, 5] The problem of unnecessary antibiotic use among children is a concern for antibiotic resistance in low- and middle-income developing countries [4–7].

There are many reasons for inappropriate antibiotic use in children. In some cases, it may be due to the fact that families have an infection that should have used antibiotics before, and then, when a similar viral infection develops, it will not be cured without antibiotics [8]. In addition, antibiotics are started even in cases where the infection is thought to be viral due to the poor geographical conditions of the area where the physician works, lack of equipment, the fear that the patient will not be able to reach the physician easily or the sick child will not be brought to control [9–11]. Furthermore, the indifference of parents in pediatric patients and the pressure to prescribe antibiotics on pediatrician also lead to inappropriate antibiotic use [12]. As a result, the administration of antibiotics provides minimal benefit, even harm (side effects, resistance, etc.) [13, 14].

Many studies have reported the relationship between antibiotic use and the development of resistance [15–17]. Turkey is one of the world’s countries with the highest consumption of antibiotics is located in European regions outside the European Union [18]. The latest report from the Organisation for Economic Co-operation and Development (OECD) Health Policy Studies, in 2015, the highest rates of antimicrobial resistance (Turkey, Korea and Greece in about 35%) of the lowest rates among the member countries stated that seven times higher [19].

Turkey has two main antimicrobial stewardship program created by the Ministry of Health. The first targets the hospitals, the second the society. The antimicrobial stewardship program targeting the community includes a 4-year (2014–2017) National Action Plan for Rational Drug Use, with an emphasis on antimicrobials at the community level. The aim is to reduce antimicrobial prescriptions, especially prescriptions for acute respiratory infections, in primary care. In this plan, it is aimed to inform doctors, pharmacists and the public on rational drug use. The sale of antibiotics without a prescription is prohibited in pharmacies. Antibiotics can only be prescribed by the doctor [20, 21]. However, various studies are needed to determine whether the National Action Plan is effective or not. Among these, the knowledge, attitudes and behaviors of parents about the use of antibiotics are of great importance. There are limited studies on this subject in Turkey and is made in a limited participant [22, 23].

This study aims to evaluate the knowledge and attitudes of parents of children with upper respiratory tract infections regarding antibiotic use and their antibiotic administration practices in a tertiary care hospital in Turkey.

## Methods

### Study area and study design

Our study is a cross-sectional survey study. It was carried out between 14 December 2021 and 1 April 2021 for parents over 18 years of age with a child under 18 years-old who applied to the general pediatrics outpatient clinics of Gazi University Faculty of Medicine Hospital Department of Pediatrics.

### Data collecting

A pilot study was conducted among 30 participants in order to check the clarity and readability of the questionnaire. The pilot test consisted of a 30-question questionnaire to identify any problems with cultural relevance, question wording, layout, and comprehension, or to gauge a respondent’s response. The results did not appear to cause uncertainty that could affect the interpretation of the questionnaire. As a result, no adjustments were made to the final survey based on the pilot test results. A face-to-face questionnaire containing 30 questions, which will take approximately 10–15 min, was given to parents. The questionnaire was administered to the parents by the clinical pharmacist in the pediatrician’s waiting room. The questionnaire was answered by only one parent when they came with the child for counseling.

Questionnaire consist of questions about parents’ demographic information, antibiotic knowledge, attitudes and practices. The demographic information of the participants from 1 to 9 of the questions was measured. The questionnaire includes questions about the knowledge level of the participant from 9 to 18, attitude from 18 to 23 and behavior from 23 to 30. Some questions were arranged from the questionnaire prepared by Panagakou et al. [24].

### Data analysis

Data were collected by paper-based questionnaires and data were entered and analyzed using Statistics Package for Social Sciences (SPSS) version 20.0 (IBM Corp., Armonk, NY, USA) for Windows. Demographic variables and answers given to knowledge, attitude and practice questions were analyzed using descriptive statistics. All of variables are categorical variables, expressed as a percentage. Chi-square test was used to compare categorical variables.

The five-point Likert scale “strongly agree” and “agree”, “strongly disagree” and “disagree” and “uncertain” were used to measure the participant’s knowledge and attitude. Practices-related questions were evaluated using the five-point Likert scales scoring scheme: “never”, “rarely”, “sometimes”, “often” and “always”. Average scores range from 1 to 5, with lower scores indicative of positive results (1 represents the best possible outcome within the domain, while 5 represents the least intended outcome). Participants scoring lower than the median were rated as “better knowledge”, “more appropriate attitude” and “better practices” about antibiotic use. The level of significance (α) was set at 0.05 for all statistical tests. Cronbach’s alpha was calculated to evaluate the degree of relationship (internal consistency) between items for each of the three domains. Cronbach alpha scores, respectively, knowledge (0.68), attitudes (0.54) and practices (0.59).

### Ethical consideration

Ethical approval for this study was obtained from ethics committee Gazi University Medical Faculty Hospital (approved numbered and date was 820 / 07.12.2020).

## Results

The questionnaires were distributed to 600 parents, but 554 parents (92.3%) responded to the questionnaire. The majority of the parents were mothers (65.5%) and the age range of the parents (60.8%) was mostly between the ages of 30–44. 25.1% of the parents were university graduates. Most of the parents (90.3%) lived in the urban areas and the income level of approximately 54.7% of them was equal to the expenditure level. Approximately half (49.5%) had a single child and 46.4% were not used antibiotics in the last year. The socio-demographic characteristics of the parents are shown in Table 1.

**Table 1:** Socio-demographic characteristics of respondents

Variables	n (%)
Gender	
Male	191 (34.5)
Female	363 (65.5)
Age (years)	
18–29	137 (24.7)
30–44	337 (60.8)
≥ 45	80 (14.4)
Education level	
İliterate	5 (0.9)
Primary school	97 (17.5)
Secondary school	120 (21.7)
High school	181 (32.7)
University	139 (25.1)
Postgraduate	12 (2.2)
Residency	
Urban	500 (90.3)
Rural	54 (9.7)
Income rate	
Less than income	152 (27.4)
Income is equivalent to expenses	303 (54.7)
More than income	99 (17.9)
Number of children	
1	274 (49.5)
2	232 (41.9)
≥ 3	48 (8.7)
Age of children	
< 1	107 (19.3)
1–6	189 (34.1)
7–11	136 (24.5)
12–14	70 (12.6)
15–17	52 (9.4)
The number of antibiotics used in the last 1 year	
0	257 (46.4)
1	180 (32.5)
2	68 (12,3)
3	27 (4.9)
≥ 4	22 (4)

### Knowledge

Parents defined “use 3 times a day “as 83.8% “every 8 hours “, 12.1% as “with main meals “, 4.2% as “at any time”. Table 2 demonstrates the responses to questions related to knowledge. A total of 15.7% of parents agreed to use antibiotics in any child with fever. 37% of the parents considered that antibiotics could cure the infections caused by the viruses. 6.3% of those who agreed with the statement of using antibiotics for prevention before getting sick, and 29.6% believed that their child would recover faster by antibiotics. 6.5% of the parents believed that antibiotics had no side effects and 6.3% believed that the effect would increase as the price of antibiotics increases. While 28% of the parents who thought that the use of inappropriate antibiotics would not change the effect and resistance of the treatment, 41% thought that new antibiotics could be developed continuously.

**Table 2:** Parental knowledge regarding antibiotic use in children with URTIs

Variables	Item	n (%)
Antibiotics can be used for any child with a fever.	Strongly disagree	172 (31)
	Disagree	157 (28.3)
	Uncertain	138 (24.9)
	Agree	71 (12.8)
	Strongly agree	16 (2.9)
Antibiotics can cure upper respiratory tract infections caused by viruses.	Strongly disagree	75 (13.5)
	Disagree	83 (15)
	Uncertain	191 (34.5)
	Agree	188 (33.9)
	Strongly agree	17 (3.1)
Children with flu-like symptoms recover faster when given antibiotics.	Strongly disagree	97 (17.5)
	Disagree	168 (30.3)
	Uncertain	125 (22.6)
	Agree	149 (26.9)
	Strongly agree	15 (2.7)
Using antibiotics in children before they get sick can prevent colds.	Strongly disagree	224 (40.4)
	Disagree	226 (40.8)
	Uncertain	64 (11.6)
	Agree	28 (5.1)
	Strongly agree	12 (2.2)
Antibiotics do not have any side effects.	Strongly disagree	236 (42.6)
	Disagree	222 (40.1)
	Uncertain	60 (10.8)
	Agree	23 (4.2)
	Strongly agree	13 (2.3)
As the price of antibiotics increases, its effect increases.	Strongly disagree	289 (52.2)
	Disagree	196 (35.4)
	Uncertain	34 (6.1)
	Agree	26 (4.7)
	Strongly agree	9 (1.6)
Inappropriate use of antibiotics does not alter the effectiveness of treatment and does not increase bacterial resistance.	Strongly disagree	116 (20.9)
	Disagree	140 (25.3)
	Uncertain	143 (25.8)
	Agree	119 (21.5)
	Strongly agree	36 (6.5)
Scientists can always produce new antibiotics for resistant bacteria.	Strongly disagree	36 (6.5)
	Disagree	78 (14.1)
	Uncertain	213 (38.4)
	Agree	196 (35.4)
	Strongly agree	31 (5.6)

Female gender compared to male gender (*p* < 0.001), high education level compared to low level (*p* = 0.006), higher income level compared to low income level (*p* = 0.047), using a small number of antibiotics in the last 1 year compared to using a large number of antibiotics in the last 1 year (*p* = 0.034) showed a better level of knowledge (Table 4).

### Attitude

**Fig. 1:**
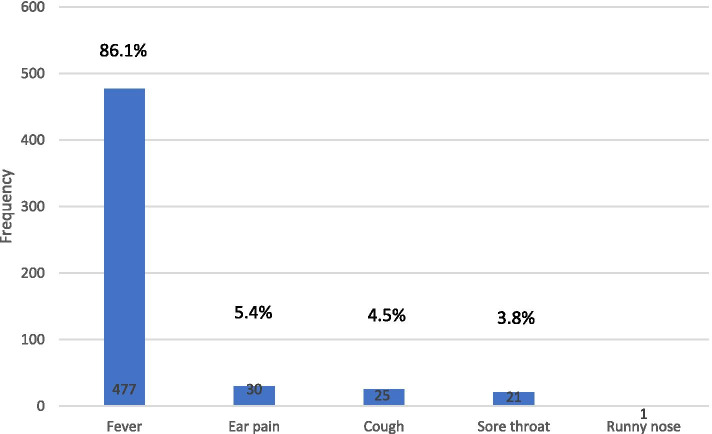
Parent’s attitude towards the most serious symptom that should be present to take their child to the doctor

**Fig. 2:**
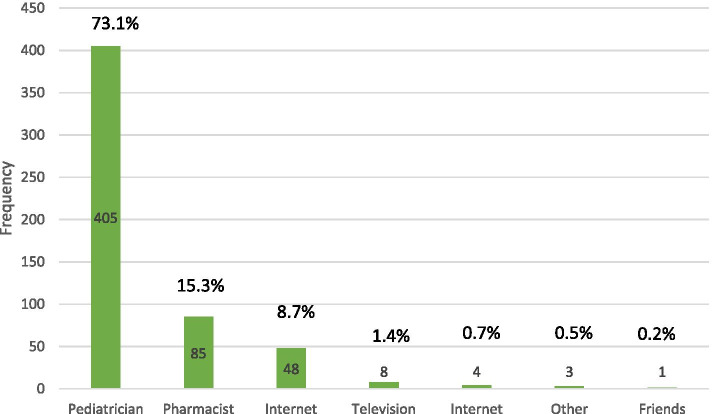
Parents source of information about the use of antibiotic treatment of the URTIs in children

Parents believed that the most important symptom (86.1%) to take their children to the pediatrician was fever, followed by ear pain (5.4%), cough (4.5%), sore throat (3.8%) and runny nose (0.2%) (Fig. 1). Parents learned about antibiotic treatment due to a URTIs by 73.1% from a pediatrician, 15.3% from a pharmacist, and 8.7% from the internet (Fig. 2). Table 3 demonstrates the responses to questions related to attitude. 64.6% of parents believed that antibiotics were overused. 80.9% of the parents declared that they thought that both parents and pediatricians should be educated about the correct use of antibiotics. 6.3% of parents put pressured their pediatrician to prescribe antibiotics.

**Table 3:** Parental attitude regarding antibiotic use in children with URTIs

Variables	Item	n (%)
I think antibiotics are overused.	Strongly disagree	39 (7)
	Disagree	75 (13.5)
	Uncertain	82 (14.8)
	Agree	287 (51.8)
	Strongly agree	71 (12.8)
I think both pediatricians and parents should get information about the correct use of antibiotics.	Strongly disagree	38 (6.9)
	Disagree	39 (7)
	Uncertain	29 (5.2)
	Agree	279 (50.4)
	Strongly agree	169 (30.5)
I put pressure on your pediatrician to prescribe antibiotics.	Strongly disagree	286 (51.6)
	Disagree	201 (36.3)
	Uncertain	32 (5.8)
	Agree	18 (3.2)
	Strongly agree	17 (3.1)

Attitudes to antibiotic use was significantly associated with education level (*p* < 0.001), areas of residence (*p* = 0.014), income level (*p* < 0.001), number of children (*p* = 0.037), age of children (*p* = 0.009) (Table 4). Those living in the urban area, those with a high education level, those with a high income level, the number of children and those with younger children had better attitudes than those living in the rural area, those with a low level of education, low income, many children and older children.

**Table 4:** Responses to questions related to knowledge, attitudes and practices in relation to antibiotics use

Variables	Knowledge Level			Attitude Level			Practices Level		
	Less (%)n	Better (%)n	p	Less (%)n	Better (%)n	p	Less (%)n	Better (%)n	p
Gender									
Male	106 (55.5)	85 (44.5)	0.001	33 (17.3)	158 (82.7)	0,823	55 (28.8)	136 (71.2)	0.011
Female	150 (41.3)	213 (58.7)		60 (16.5)	303 (83.5)		70 (19.3)	293 (80.7)	
Age group									
18–29	61 (44.5)	76 (55.5)	0.233	18 (13.1)	119 (86.9)	0,074	291 (21.2)	108 (78.8)	0.133
30–44	151 (44.8)	186 (55.2)		55 (16.3)	282 (83.7)		71 (21.1)	266 (78.9)	
≥ 45	44 (55)	36 (45)		20 (25)	60 (75)		25 (31.2)	55 (68.8)	
Education level									
İliterate	3 (60)	2 (40)	0.006	2 (40)	3 (60)	< 0.001	2 (40)	3 (60)	0.953
Primary school	52 (53.6)	45 (46.4)		31 (32)	66 (68)		22 (22.7)	75 (77.3)	
Secondary school	61 (50.8)	59 (49.2)		24 (20)	96 (80)		27 (22.5)	93 (77.5)	
High school	91 (50.3)	90 (49.7)		24 (13.3)	157 (86.7)		41 (22.7)	140 (77.3)	
University	45 (32.4)	94 (67.6)		12 (8.6)	127 (91.4)		31 (22.3)	108 (77.7)	
Postgraduate	4 (33.3)	8 (66.7)		0 -	12 (100)		2 (16.7)	10 (77.3)	
Residency									
Urban	228 (45.6)	272 (54.4)	0.381	77 (15.4)	423 (84.6)	0.014	108 (21.6)	392 (78.4)	0.099
Rural	28 (51.9)	26 (48.1)		16 (29.6)	38 (70.4)		17 (31.5)	37 (68.5)	
Income rate									
Less than income	77 (50.7)	75 (49.3)	0.047	26 (17.1)	126 (82.9)	< 0.001	37 (24.3)	115 (75.7)	0.029
Income is equivalent to expenses	144 (47.5)	159 (52.5)		50 (16.5)	253 (83.5)		57 (18.8)	246 (81.2)	
More than income	35 (35.4)	64 (64.6)		17 (17.2)	82 (82.8)		31 (31.3)	68 (68.7)	
Number of children									
1	119 (43.4)	155 (56.6)	0.425	39 (14.2)	235 (85.8)	0.037	54 (19.7)	220 (80.3)	0.098
2	113 (48.7)	159 (51,3)		40 (17.2)	192 (82.8)		55 (23.7)	177 (76.3)	
≥ 3	24 (50)	24 (50)		14 (29.2)	34 (70.8)		16 (33.3)	32 (66.7)	
Age of children									
< 1	50 (46.7)	57 (53.3)	0.659	14 (13.1)	93 (86.9)	0.009	25 (23.4)	82 (76.6)	0.961
1–6	84 (44.4)	105 (55.6)		22 (11.6)	167 (88.4)		41 (21.7)	148 (78.3)	
7–11	68 (50)	68 (50)		28 (20.6)	108 (79.4)		33 (24.3)	103 (75.7)	
12–14	28 (40)	42 (60)		13 (18.6)	57 (81.4)		14 (20)	56 (80)	
15–17	26 (50)	26 (50)		16 (30.8)	36 (69.2)		12 (23.1)	40 (76.9)	
The number of antibiotics used in the last 1 year									
0	114 (44.4)	143 (55.6)	0.034	36 (14)	221 (86)	0.356	40 (15.6)	217 (84.4)	< 0.001
1	75 (41.7)	105 (58.3)		32 (17.8)	148 (82.2)		43 (23.9)	137 (76.1)	
2	43 (63.2)	25 (36.8)		16 (23.5)	52 (76.5)		26 (38.2)	42 (61.8)	
3	12 (44.4)	15 (55.6)		4 (14.8)	23 (85.2)		8 (29.6)	19 (70.4)	
≥ 4	12 (54.5)	10 (45.5)		5 (22.7)	17 (77.3)		8 (36.4)	14 (63.6)	

### Practice

**Fig. 3:**
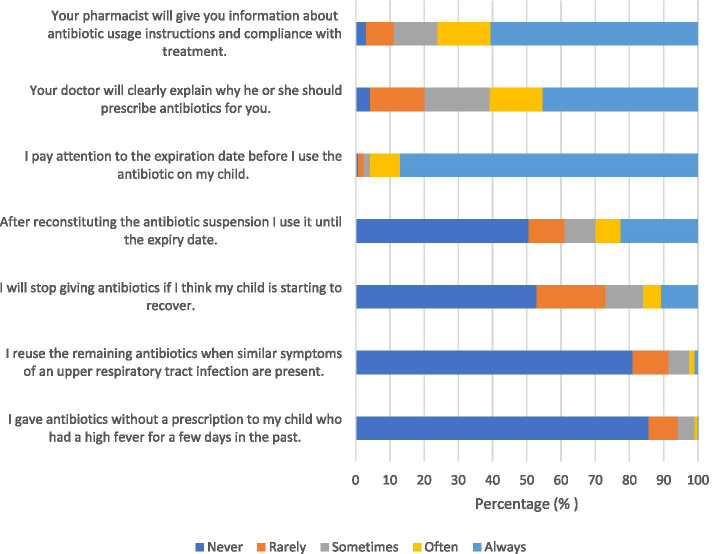
Parents responses (%) to questions related to practice

65.7% of the parents kept the antibiotic suspension in the refrigerator, 31.8% in the medicine cabinet, and 2.3% in anywhere. Figure 3 demonstrates the responses to questions related to practice. 85.6% of the parents declared that they never gave their children antibiotics without a prescription when they had a fever. 80.9% declared that they never reused old antibiotics in the presence of a new infection. 52.9% of them stated that they never stopped using antibiotics when they thought their children were getting better. 50.5% of the parents declared that they never used the antibiotic suspension until the expiry date after reconstitution and 86.8% always paid attention to the expiration date of the antibiotics.

45.3% of the parents believed that pediatricians always explained the reason for prescribing antibiotics, and 60.5% of the parents stated that they always received information from their pharmacists about antibiotic usage instructions and compliance with treatment. Female gender compared to male gender (*p* = 0.011), higher income level compared to low income level (*p* = 0.029), lower number of antibiotics used in the last 1 year compared to higher number of antibiotics used in the last 1 year (*p* < 0.001) were associated with better practice (Table 4).

## Discussion

In our study, we assessed knowledge, attitudes and practices on antibiotic treatment of parents in Turkey. It is the most comprehensive and current study on this subject in Turkey. Our study showed that parents have a good attitude and practice in the use of antibiotics. A total of 37.7% of parents agreed to use antibiotics in any child with fever. While the rate of agreeing with this statement was 72.4% in a study conducted in Jordan [14], it was found to be lower than 10% in the study conducted in Greece [24]. In a study conducted in Italy, 92.9% of respondents knew that antibiotics did not have a direct effect on fever [25]. Parents agreed with 37% that antibiotics could be used in the treatment of infections caused by viruses. While 59% of Palestinian parents and 17.2% of Croatian parents agreed agreed with this idea, Greek parents thought that 80% of the viruses were spontaneously limited and did not use antibiotics [5, 24, 26]. Those who agreed with the statement of using antibiotics for prevention before getting sick were 7.3%. This was also a very low rate. Three times a day use was correctly defined 83.8% as “every 8 h’. On the contrary, Rowa’J et al. [27] in their study defined only 28% as “every 8 h” and 56% with “the main meals”. In our study, parents believed that the most important symptom (86.1%) to take their children to the pediatrician was fever. Parents from Jordan, Palestine and Malaysia [5, 14, 28] agreed with this view, while parents in Greece and Minnesota expected antibiotics to be prescribed for ear pain [24, 29].

In our study, we found that female gender was associated with better knowledge and practice level than male gender. However, this may be due to the fact that female gender constituted a large part of the sample.

According to the results of our study, most of the parents (73.1%) learned the data about antibiotics from the pediatricians, and very few (6.3%) put pressure on the pediatricians to prescribe antibiotics. This situation showed that the parents trusted the pediatrician.

Parents generally showed appropriate behavior in practice. 85.6% of the parents stated that they never gave antibiotics without a prescription to their children with fever. 80.9% of them stated that they never reused the old antibiotic again in the presence of a new infection. It was a very positive result compared to studies conducted in other countries [5, 14, 30]. This may be related to the increasing awareness of parents about rational antibiotic use. The impact of the national action plan on rational drug use can be great in this situation. Within the scope of this action plan, messages about rational drug use were given on television, in news programs, in social media and even in TV series. Posters about the rational use of antibiotics were hung where they could be seen by the public. This may have caused parents to use antibiotics rationally.

Most of the parents declared that (86.3%) always paid attention to the antibiotic expiration date. However, 50.5% of parents declared that they never used the antibiotic suspension until the expiration date after reconstitued it. This meant that about half of them gave the correct answer, as the antibiotic suspension should be consumed within a maximum of 10 days [27]. But Rowa’J et al. [27] showed that 98.5% of mothers applied it correctly. In our study female gender compared to male gender, high education level compared to low level, higher income level compared to low income level, using a small number of antibiotics in the last 1 year compared to using a large number of antibiotics in the last 1 year showed a better level of knowledge. In the study of Al Saleh et al. [31] female gender, high education level and surprisingly low income level were found to be associated with better knowledge level. High level of education, living in the urban areas, high level of income, low number of children, younger children were associated with better attitude level, while Al Saleh et al. [31] found higher income and better attitude levels in more than 3 children. In our study female gender compared to male gender, higher income level compared to low income level, lower number of antibiotics used in the last 1 year compared to higher number of antibiotics used in the last 1 year were associated with better practice. Hernandez et al. [32] showed in a multivariable analysis that the level of education and the low number of antibiotics used in 1 year were associated with a better level of knowledge and behavior.

### Strengths and limitations of the study

In our study, 92.3% of people responded to the questionnaire. This is a very good rate. Furthermore, this study is a comprehensive study to assess knowledge, attitudes and practices of parents in Turkey about the treatment and the use of antibiotics. However, this study has some limitations. This study was conducted in a tertiary hospital in the capital of Turkey. Although there were applicants from every region of Turkey to the this hospital; our sample may not represent the entire community in Turkey*.* Our study reflects current parents’ knowledge, attitude and practice. Unfortunately, we do not know the knowledge, attitudes and practices of parents before the national action plan for rational drug use came into effect. National action plans on rational drug use should be continued in the future. Before and after the national action plan, studies involving parents’ knowledge, attitudes and practices should be carried out.

## Conclusions

According to the results of our study of parents’ lack of knowledge about antibiotics in Turkey, though generally it shows proper attitude and behavior. It shows that some of the restrictions imposed by the National Action Plan are partially working. Among these restrictions, the fact that antibiotics are prescribed only by a doctor and sold only in pharmacies limited self-use without a prescription and reduced antibiotic consumption. However, it is still necessary to keep parents, pediatricians and pharmacists informed about the use of antibiotics and to be more careful about the prescribing of antibiotics, and if necessary, sanctions should be imposed by the state to prevent unnecessary antibiotic prescriptions.

## Data Availability

All data generated or analyzed during this study are included in this published article.

## Abbreviations

URTIs: Upper respiratory tract infections
OECD: Organisation for Economic Co-operation and Development

## References

[1] Eckel N, Sarganas G, Wolf I-K, Knopf H (2014). Pharmacoepidemiology of common colds and upper respiratory tract infections in children and adolescents in Germany. BMC Pharmacol Toxicol.

[2] Zeng L, Zhang L, Hu Z, Ehle EA, Chen Y, Liu L (2014). Systematic review of evidence-based guidelines on medication therapy for upper respiratory tract infection in children with AGREE instrument. PLoS One.

[3] Nyquist A-C, Gonzales R, Steiner JF, Sande MA (1998). Antibiotic prescribing for children with colds, upper respiratory tract infections, and bronchitis. JAMA..

[4] Hoa NQ, Chuc NTK, Phuc HD, Larsson M, Eriksson B, Lundborg CS (2011). Unnecessary antibiotic use for mild acute respiratory infections during 28-day follow-up of 823 children under five in rural Vietnam. Trans R Soc Trop Med Hyg.

[5] Sa’ed HZ, Taha AA, Araj KF, Abahri IA, Sawalha AF, Sweileh WM (2015). Parental knowledge, attitudes and practices regarding antibiotic use for acute upper respiratory tract infections in children: a cross-sectional study in Palestine. BMC Pediatr.

[6] World Health Organization. Antimicrobial resistance: global report on surveillance 2014. Geneva: WHO; 2014.

[7] Yang Y-H, Fu S, Peng H, Shen A-D, Yue S, Go Y (1993). Abuse of antibiotics in China and its potential interference in determining the etiology of pediatric bacterial diseases. Pediatr Infect Dis J.

[8] Currie J, Lin W, Zhang W (2011). Patient knowledge and antibiotic abuse: evidence from an audit study in China. J Health Econ.

[9] Paluck E, Katzenstein D, Frankish CJ, Herbert CP, Milner R, Speert D (2001). Prescribing practices and attitudes toward giving children antibiotics. Can Fam Physician.

[10] Butler CC, Rollnick S, Pill R, Maggs-Rapport F, Stott N (1998). Understanding the culture of prescribing: qualitative study of general practitioners' and patients' perceptions of antibiotics for sore throats. BMJ..

[11] Lopez-Vazquez P, Vazquez-Lago JM, Figueiras A (2012). Misprescription of antibiotics in primary care: a critical systematic review of its determinants. J Eval Clin Pract.

[12] Souto-López L, Vazquez-Cancela O, Vazquez-Lago JM, López-Durán A, Figueiras A (2020). Parent-related factors influencing antibiotic use in a paediatric population: A qualitative study in Spain. Acta Paediatr.

[13] Stivers T (2002). Participating in decisions about treatment: overt parent pressure for antibiotic medication in pediatric encounters. Soc Sci Med.

[14] Hammour KA, Jalil MA, Hammour WA (2018). An exploration of parents’ knowledge, attitudes and practices towards the use of antibiotics in childhood upper respiratory tract infections in a tertiary Jordanian hospital. Saudi Pharm J.

[15] Shibl A, Memish Z, Osoba A (2001). Antibiotic resistance in developing countries. J Chemother.

[16] Barbosa TM, Levy SB (2000). The impact of antibiotic use on resistance development and persistence. Drug Resist Updat.

[17] Goossens H (2009). Antibiotic consumption and link to resistance. Clin Microbiol Infect.

[18] Versporten A, Bolokhovets G, Ghazaryan L, Abilova V, Pyshnik G, Spasojevic T (2014). Antibiotic use in eastern Europe: a cross-national database study in coordination with the WHO regional Office for Europe. Lancet Infect Dis.

[19] Isler B, Keske Ş, Aksoy M, Azap Ö, Yilmaz M, Yavuz S (2019). Antibiotic overconsumption and resistance in Turkey. Clin Microbiol Infect.

[20] WHO Regional Office for Europe. Turkey takes strong action to reduce antibiotic consumption and resistance. Copenhagen: World Health Örganization Regional Öffice for Europe; 2017. http://www.euro.who.int/en/health-topics/disease-prevention/antimicrobialresistance/news/news/2017/11/turkeytakes-strong-action-to-reduce-antibioticconsumption-and-resistance; Erişim tarihi: 01.09.2020.

[21] Aksoy M, Alkan A, Isli F (2015). Rational drug use promotional activities of Ministry of Health. Turkiye Klinikleri J Pharmacol-Special Topics.

[22] Korkut Y, Alime E, Ayada C (2019). Evaluation of the knowledge, attitudes, and behaviors of antibiotics usage at the parents living in the Aegean part of Turkey. Konuralp Med J.

[23] Bayram N, Günay İ, Apa H, Gülfidan G, Yamaci S, Kutlu A (2013). Evaluation of the factors affecting the attitudes of the parents towards to use of antibiotics/Çocuklarda Antibiyotik Kullanimi ile Ilgili Ailelerin Tutumlarini Etkileyen Faktörlerin Degerlendirilmesi. Cocuk Enfeksiyon Derg.

[24] Panagakou SG, Spyridis Ν, Papaevangelou V, Theodoridou KM, Goutziana GP, Theodoridou MN (2011). Antibiotic use for upper respiratory tract infections in children: a cross-sectional survey of knowledge, attitudes, and practices (KAP) of parents in Greece. BMC Pediatr.

[25] Pierantoni L, Lo Vecchio A, Lenzi J, Corsi V, Campana L, Luca Trobia G (2021). Parents’ perspective of antibiotic usage in children: A Nationwide survey in Italy. Pediatr Infect Dis J.

[26] Farkaš M, Glažar Ivče D, Stojanović S, Mavrinac M, Mićović V, Tambić AA (2019). Parental knowledge and awareness linked to antibiotic use and resistance: comparison of urban and rural population in Croatia. Microb Drug Resist.

[27] Rowa’J A-R, Anabousi H. (2015). Problems associated with reconstitution, administration, and storage of antibiotic suspensions for pediatrics: a cross-sectional study in Nablus city, Palestine. BMC Res Notes.

[28] Chan G, Tang S (2006). Parental knowledge, attitudes and antibiotic use for acute upper respiratory tract infection in children attending a primary healthcare clinic in Malaysia. Singap Med J.

[29] Belongia EA, Naimi TS, Gale CM, Besser RE (2002). Antibiotic use and upper respiratory infections: a survey of knowledge, attitudes, and experience in Wisconsin and Minnesota. Prev Med.

[30] Albalawi GA, Abdulrahman Alzahrani S, Alotaibi AA, Ghmaird AS (2020). Knowledge, attitude, and practices of parents regarding the use of antibiotics among their children with upper respiratory tract infections. IJMDC.

[31] Al-Saleh S, Abu Hammour K, Abu HW (2020). Influencing factors of knowledge, attitude, and practice regarding antibiotic use in children with upper respiratory tract infections in Dubai. J Eval Clin Pract.

[32] Hernández-Díaz I, Ayala-Meléndez A, González-González E, Rosario-Calderón I, Figueroa-Ríos D, Melin K (2019). Knowledge and beliefs, behaviors, and adherence among Latino parents or legal guardians related to antibiotic use for upper respiratory tract infections in children under 6 years of age. J Am Pharm Assoc.

